# Weight-height relationships and central obesity in 7-year-old to 10-year-old Polish urban children: a comparison of different BMI and WHtR standards

**DOI:** 10.1186/s40101-015-0073-3

**Published:** 2015-10-07

**Authors:** Paweł Tomaszewski, Piotr Żmijewski, Katarzyna Milde, Edyta Sienkiewicz-Dianzenza

**Affiliations:** Department of Statistics and Computer Science, Jozef Pilsudski University of Physical Education, Marymoncka 34, 00-968 Warsaw, Poland; Department of Physiology, Institute of Sport, Trylogii 2, 01-982 Warsaw, Poland

**Keywords:** Weight-height relationships, Central obesity, Classification consistency, Children

## Abstract

**Background:**

An increase in overweight and obesity rates among children is a major social problem; however, interpretation and comparability of estimations may be affected by the reference values and cut-off points used. The aim of the study was to assess the prevalence of underweight, overweight, obesity and central obesity in 7-year-old to 10-year-old urban children and to compare the results obtained through various standards of BMI or waist-to-height ratio (WHtR) indicators.

**Methods:**

The research was conducted on a sample group of 367 girls and 424 boys aged 6.5–10.5 years, randomly chosen from a number of primary schools in Warsaw, Poland. In all participants, basic somatic features were assessed, and based on BMI and WHtR values, participants were then classified according to different standards. The prevalence of underweight, overweight, obesity and central obesity in boys and girls was compared using the chi-square test; fractions obtained from various BMI and WHtR standards were compared through a test for proportions; and the conformity of classification methods was assessed using Cohen’s kappa coefficient.

**Results:**

Approximately 9 % of girls and 6 % of boys were underweight, 15 % of all participants was classified as overweight, and approximately 4 % of girls and 6 % of boys aged 7–10 were obese. Central obesity was diagnosed in 18.6–20.9 % of all participants, while another 7.1 % of girls and 7.5 % of boys displayed symptoms of excessive fat deposition characterized by elevated body fat percentages. Even though the prevalence estimations varied depending on the standards used, the overall classification compliance reached 86–94 % with a Cohen’s kappa coefficient ranging from 0.676 to 0.841.

**Conclusion:**

The prevalence of underweight, overweight and obesity among urban children is comparable to estimates for the general population. Of particular concern, in terms of health and proper physical development, is the problem of central obesity that affects one out of five children. The use of reference values representing body fat percentage seems justified as it allows for a more precise diagnosis of weight-related disorders, including the particularly threatening abdominal obesity.

## Background

The steady increase in obesity rates among children and teenagers has become a major social problem, reaching the size of an epidemic in most developed countries [1–3]. Research indicates that one in every five children living in Europe is either overweight or obese, and around 400,000 new cases of obesity or overweight are diagnosed each year among school children [4]. At the same time, there is more and more emphasis on the fact that untreated obesity during childhood frequently causes major health problems in adulthood [5, 6], giving rise to hypertension, type 2 diabetes, dyslipidaemia, metabolic syndrome or ischemic heart disease [7, 8]. In most cases, these medical conditions will significantly decrease the patients’ quality of life and will require treatment for the majority of their adult lives [9]. Hence, the issues related to overweight and obesity and the frequency of their occurrence remain a significant problem in the eyes of most medical researchers.

In practical terms, the preliminary diagnosis of overweight and obesity in children is conducted with the use of well-known anthropometric indicators. One of the most widely used indicators is the body mass index (BMI). However, this method is somewhat restrictive in its uses. The limited applicability of the BMI in measuring body fat in children and adolescents has been demonstrated in the past [10, 11]. Other increasingly popular methods for measuring body fat and abdominal obesity are the waist circumference (WC) or the waist-to-height ratio (WHtR), which turn out to be particularly strong predictors of cardiovascular diseases compared to BMI [11–13].

The problem with using the BMI system for diagnosing underweight, overweight and obesity and for other comparative studies between populations is compounded by the fact that researchers frequently apply different reference values and cut-off points for the assessment of weight-to-height ratio disorders. The BMI-based diagnosis is usually performed using percentile norms and cut-off points specifically determined for a given population, where the most commonly used cut-off points are the <5, >85 and >95 percentiles for underweight, overweight and obesity. This approach is useful for estimating the prevalence of obesity and overweight among the general population, but it proves largely ineffective for making comparisons between countries. Based on larger international studies, the Obesity Task Force organization proposed a unified definition for diagnosing underweight, overweight and obesity in children and young adults aged 2–18 [14, 15], which adopts diagnostic criteria similar to those used for the adult population. The proposed reference values have gained many supporters, but they also have sparked a large-scale discussion regarding the applicability of international and national standards pointing to the fact that national standards are usually more reliable in examining a given population [16, 17]. A similar problem occurs in the case of diagnosing central obesity, which is commonly based on the correlation between the WHtR index and a cut-off point of 0.5 for a given age and sex. Since the WHtR index and the fixed cut-off points (0.5) used for both sexes are often considered unreliable for assessing individuals during stages of growth [18], this study uses additional cut-off points in diagnosing central obesity that are specifically designed for the Polish population and account for the participants’ age and sex [19]. The aim of this study was to determine the prevalence of underweight, overweight, obesity and central obesity in 7-year-old to 10-year-old urban children as assessed by different BMI and WHtR standards.

## Methods

The test group consisted of 367 girls and 424 boys aged 6.5–10.5, randomly chosen from a number of primary schools located in various districts of Warsaw, Poland. The numbers of girls and boys within individual age categories were respectively 61 and 64 for 7-year-olds, 93 and 123 for 8-year-olds, 127 and 144 for 9-year-olds, and 86 and 93 for 10-year-olds. Basic somatic features were measured for all participants using standard methodologies and devices [20]. All measurements were taken during physical fitness classes and by the same trained personnel. Both the children and their parents were informed about the purpose and form of the study and gave their informed consent. The study was approved by the local Ethics and Clinical Research Committee and performed according to the indications contained in the Declaration of Helsinki.

The height of all test participants was measured with an anthropometer, with accuracy within 0.1 cm; their body mass was estimated using the body composition analyser TANITA TBF-300A (Japan) with accuracy within 0.1 kg. A skinfold calliper was used to measure the thickness of three distinct skinfolds around the body (triceps, subscapula, suprailiac) with accuracy within 0.5 mm. Waist circumference was measured at naval height while patients held their breath. The measurements obtained were used to calculate the BMI and WHtR values and to assess the body fat percentage (%FAT) according to the Slaughter equation [21].

The BMI values obtained were used to divide participants into groups depending on their weight-to-height ratios, according to the following criteria:

1. The international standard of the International Obesity Task Force (IOTF)—the classification included cut-off points adjusted for the age and sex of participants in diagnosing underweight [15], as well as overweight and obesity [14].
2. BMI percentile value tables adjusted for specific age and sex groups developed on the basis of a Polish nationwide study of children and teenagers in 2010 [22]. According to the guidelines provided by the Centers for Disease Control and Prevention [23], a BMI in the ≤5 percentile indicates underweight, a BMI in the ≥85 percentile indicates overweight and a BMI in the ≥95 percentile indicates obesity.
3. BMI reference values developed on the basis of population data on individuals having a correct body fat percentage [24]. The classification of participants was performed using polynomial equations as functions of age computed for boys and girls and BMI cut-off points corresponding to the World Health Organization’s (WHO) recommended values [25], i.e. BMI < 18.5 underweight, BMI > 25 overweight and BMI > 30 obesity.

In addition, the prevalence of central (abdominal) obesity was assessed in all participants using the standard WHtR ≥ 0.5 criteria [26] and WHtR cut-off points representing age, sex and the estimated body fat percentage [19]. In the latter method, the logarithmic equations were used to standardize WHtR values recorded in all participants. Those with estimated excessive body fat content were further classified with respect to age- and gender-specific reference WtHR values defined as mean ± 2 SD. A WtHR value within normal limits was considered to indicate relatively uniform, peripheral fat distribution while values above the upper limit indicated central (abdominal) fat deposition.

The prevalence of central obesity in boys and girls and individual BMI categories were compared through a chi-square test. The differences in percentage values obtained from various BMI and WHtR standards were assessed through a test for proportions. The classification algorithms were assessed by calculating the percentage of compliant classifications for each pair of methods and by determining Cohen’s kappa coefficient with 95 % confidence intervals. The values of Cohen’s kappa coefficient were interpreted according to the classification proposed by Fleiss et al. [27]. The level of statistical significance was set at *a* = 0.05.

## Results

The percentages of participants assigned to each BMI category according to the applied criteria are presented in Table 1. In general, irrespectively of the classification applied the results indicated no variability in the occurrence of underweight, overweight and obesity in boys and girls. The only significant differences were found for estimates using the IOTF criteria, where there was a lower percentage of underweight boys than girls (7.8 vs. 13.7 %; *p* < 0.01); moreover, using the criteria proposed by Tomaszewski et al. [24] indicated about a 5 % higher obesity ratio in boys than in girls (*p* < 0.01). On the other hand, the estimates based on different BMI criteria produced a higher variability rates between individual standards, e.g. the IOTF criteria indicated almost a threefold higher prevalence of underweight girls (13.7 vs. 4.7 %; *p* < 0.001) and a twofold increase in the number of underweight boys (7.8 vs. 3.8 %; *p* < 0.01) compared to the numbers estimated using cut-off points proposed by Kułaga et al. [22]. Moreover, regardless of sex, using the latter criteria the number of participants with normal body mass was about 4–11 % higher than estimated by other methods. In contrast, the criteria proposed by Tomaszewski et al. [24] suggested a slight increase in obesity or overweight ratios among boys as compared to other BMI standards. Nevertheless, the overall results of the study suggest a strong degree of compliance between the applied classification methods (Table 2). The calculated percentages of consistent classifications for pairs of classification methods reached compatibility rates of 86.5 and 93.4 %, levels that are also reflected in the high Cohen’s kappa coefficient values that ranged from 0.676 to 0.841. Therefore, generally speaking, it can be stated that the problem of underweight affects 9 % of urban girls and 6 % of urban boys, 15 % of boys and girls are overweight, and 4 % of boys and 6 % of girls aged 7–10 are obese.

**Table 1:** Percentages (SE) of examined children grouped into specific BMI categories based on different BMI standards

Category	IOTF standards^a^	Kułaga et al. standards [22]	Tomaszewski et al. standards [24]
Girls (*n* = 367)			
Underweight	13.7 (1.8)	4.7 (1.1)****	8.5 (1.5)**^,^*****
Normal	66.2 (2.5)	76.9 (2.2)***	72.8 (2.3)**
Overweight	15.7 (1.9)	13.5 (1.8)	15.7 (1.9)
Obesity	4.4 (1.1)	4.9 (1.1)	3.0 (0.9)
Boys (*n* = 424)			
Underweight	7.8 (1.3)*	3.8 (0.9)**	5.9 (1.1)
Normal	71.6 (2.2)	80.6 (1.9)**	69.5 (2.2)*******
Overweight	15.1 (1.7)	10.2 (1.5)**	16.5 (1.8)******
Obesity	5.4 (1.1)	5.4 (1.1)	8.0 (1.3)*

*Significantly different from the rate of occurrence observed in girls (*p* < 0.01). Significantly different from the percentage estimated using the IOTF criteria: **p < 0.05; ***p < 0.01; ****p < 0.001. Significantly different from the percentage estimated using the criteria proposed by Kułaga et al. [22]: *****p < 0.05; ******p < 0.01; *******p < 0.001

^a^IOTF indicates the criteria proposed by Cole et al. [14, 15]

**Table 2:** Compliance of the classification of participants using various BMI criteria

Parameter	Standard		
	IOTF^a^ vs. Kułaga et al. [22]	IOTF^a^ vs. Tomaszewski et al. [24]	Kułaga et al. [22] vs. Tomaszewski et al. [24]
Girls (*n* = 367)			
The percentage of consistent classification	88.7 %	91.5 %	93.4 %
Cohen’s kappa coefficient	0.756	0.823	0.841
95 % confidence interval	0.687–0.824	0.764–0.881	0.781–0.900
Consistency^b^	Excellent	Excellent	Excellent
Boys (*n* = 424)			
The percentage of consistent classification	91.0 %	87.7 %	86.5 %
Cohen’s kappa coefficient	0.776	0.737	0.676
95 % confidence interval	0.709–0.843	0.672–0.803	0.603–0.750
Consistency^b^	Excellent	Good	Good

^a^IOTF indicates the criteria proposed by Cole et al. [14, 15]

^b^Classification according to Fleiss et al. [27]

Central obesity was diagnosed in 21 % of the participants regardless of sex using WHtR cut-off points that included age, sex and body fat percentage. In addition, central fat distribution was also diagnosed in another 7.1 % of girls and 7.5 % of boys having elevated body fat content. The results were identical to the occurrence of central obesity in girls and slightly lower in boys (20.9 and 18.6 %, respectively) as estimated using the commonly applied criteria of WHtR ≥ 0.5. The estimated overall classification compliance was high for both WHtR standards: the percentage of consistent classifications equalled to 91.3 and 93.9 %, while the Cohen’s kappa coefficient reached 0.742 (95 % CI, 0.658–0.826) and 0.805 (95 % CI, 0.733–0.877), for girls and boys, respectively.

Interestingly, among all 77 girls classified as having central obesity (WHtR values ≥0.5), as many as 29–34 % had normal body mass depending on the adopted BMI standard while 14, 21 and 23 % were classified obese using Tomaszewski et al. [24], IOTF [14, 15] and Kułaga et al. [22] standards, respectively. In boys with diagnosed central obesity (*n* = 79), the percentages of those with BMI within normal limits varied from 8 to 23 % by Tomaszewski et al. [24] and Kułaga et al. [22] standards, respectively. The fractions of obese boys were 29 % for both the IOTF [14, 15] and Kułaga et al. [22] standards and 41 % for criteria proposed by Tomaszewski et al. [24]. On the other hand, irrespectively of the BMI standard used, all participants classified as obese using BMI criteria had WHtR values ≥0.5.

## Discussion

The results of a number of studies indicate an increasing prevalence of overweight and obesity in children, and the accelerating rate at which children of an increasingly younger age are diagnosed with excessive body weight and obesity is particularly alarming [2]. According to the latest United Nations Children’s Fund (UNICEF) report [28], Polish children are gaining weight at the fastest rate in all of Europe, and the percentage of obese children in Poland has doubled over the last decade. Considering the importance of this problem, this study attempted to assess the prevalence of underweight, overweight and obesity in 7-year-old to 10-year-old Polish urban children. Since the interpretation and comparability of the acquired results may be affected by the reference values and cut-off points assumed, the evaluation of the results was carried out using different standards and compared the statistical compliance of all estimated values.

Early detection of overweight and obesity in children and adolescents is usually based on the commonly used BMI. However, the predictive power of BMI as a measure of adiposity was considered insufficient [11, 29] mostly due to the fact that the definition of obesity refers to an excessive body fat content, the physiological risk factor, and not to the weight-height proportion. Therefore, the overweight assessed by BMI standards was shown to be insufficiently associated with an increased body fat content, resulting in discrepancies between the body fat percentage and BMI criteria in detecting overweight and obesity [30–34]. In contrast, Laurson et al. [35] have found that BMI and skinfold-derived body fat contents demonstrate reasonable agreement when used to classify adiposity status in children and adolescents. Nevertheless, using BMI alone may result in possible stigmatization of children by incorrectly classifying them as weight-deficient or obese [36]. For that reason, other indices like WC and WHtR gain popularity as both were shown to be better tools than BMI in assessing the risk of overweight- and obesity-related cardiovascular diseases [12, 13, 37, 38]. Moreover, WHtR seems especially valuable since WC alone may be imprecise in case of subjects of extreme stature [26]. It was also shown that neither WC nor BMI was predictive of risk of cardiovascular diseases if adiposity was not included as a risk factor [39] and WHtR was considered more sensitive than BMI as an early warning of health risks [40]. That is why the WHtR has been recently widely used to detect the risk of central (abdominal) obesity; the most commonly applied critical value of that index is equal to 0.50 irrespectively of age and gender, values exceeding 0.6 being considered alarming [41]. That criterion has been, however, disputed especially in the period of growth when the gains in body height and waist circumference may not parallel one another, and the use of an identical criterion for both genders has also been questioned [18]. Nevertheless, WHtR = 0.5 is a commonly considered suitable global boundary value [26].

Although the estimated rates of obesity and overweight found in this paper differed slightly between sets of criteria used, it can be concluded that the problem of excessive body weight affects about 15 % of the participants, and 6 % of boys and 4 % of girls in Warsaw aged 7 to 10 years old can be defined as obese. Moreover, irrespectively of gender, central obesity was diagnosed in about 20 % of study participants using WHtR criteria. The obtained results correlate strongly with the findings of other authors who estimate that the problem of excessive body weight affects between 15 and 22 % of Polish boys and girls aged 6–18 [22, 42]. In the study of Polish elementary school children [43], the rates of overweight and obesity among urban boys were 16.9 and 5.5 %, respectively, and 15.1 and 3.5 % in urban girls, respectively, the estimations being nearly identical to our findings. In the study of Bac et al. [44], the prevalence of overweight among Polish urban boys aged 6–13 years reached 27 % and was considerably higher than the prevalence observed in this study while the estimations for urban girls were comparable (16 %). Moreover, obesity was identified in about 8 % of urban boys and in 3.5 % of urban girls which is partly in line with our observations. The prevalence of underweight among elementary school Polish urban children amounted to 11.2 and 13.4 % in boys and girls, respectively [43], while in our study, depending on the standard used, underweight was diagnosed in 3.8–8.5 % of participants, with the exception of IOTF criteria for girls, where nearly 14 % of female participants were classified underweight.

The construction and adoption of suitable reference values that represent basic somatic features is of paramount importance in assessing a child’s development process. It is particularly important to properly define normal body weight and body fat percentages in order to effectively diagnose obesity. While the use of BMI and its criteria for diagnosing obesity is suitably suited for examining the adult population, its applicability in diagnosing children in various stages of development is still the subject of a heated discussion when it comes to the assessment of BMI indicators and accepted threshold values. This frequently causes researchers to use their own individual set of criteria in carrying out studies, which, in turn, makes the results incompatible and incomparable with those obtained by other researchers. In this study, the compliance of estimates performed with different reference values was verified using three standards based on BMI values. The three standards compared differed in terms of assumed reference values and cut-off points, as well as the methods of data selection used in developing them. The first, the IOTF standard, was developed using data from a number of international studies performed according to the lambda-mu-sigma (LMS) method and published in two other studies [14, 15]. The IOTF standard involved drafting BMI curves adjusted for age and sex of participants, passing through the 18.5, 25.0 and 30.0 values for 18-year-olds. The second standard proposed by Kułaga et al. [22] was based on data gathered from the general population of Polish school-age children (*n* > 17,500) and included BMI percentile value tables for 7-year-old to 18-year-old boys and girls. The BMI percentile value tables were developed using the LMS method. Both of the standards described above used BMI reference values based on the data coming from the general population without accounting for the body fat percentage measured in study participants. This is a considerable drawback since the BMI values do not correlate with the physiological criteria of obesity and therefore may not provide the necessary basis for making a comprehensive diagnosis [45]. Because of that, the third standard proposed by Tomaszewski et al. [24] used population data gathered only from individuals with a healthy body fat percentage (*n* > 1800). This standard utilized sex-specific BMI equations for age. Similar to the IOTF standard, this standard included BMI centile curves and cut-off points following recommendations of the WHO. As for central obesity, the estimates were tested for compliance using the widely adopted criteria of WHtR ≥ 0.5 and WHtR cut-off points which, just as in the case of BMI, were determined on the basis of population data gathered from individuals with healthy body fat percentages.

The statistical compliance tests indicate that the classifications performed both in accordance with the IOTF standard and based on the criteria proposed by Tomaszewski et al. [24] achieved the lowest level of differences in the reported estimates, ranging from 0 to 7 % depending on sex and BMI category. A slightly greater variability was observed in the percentile values calculated based on IOTF cut-off points and percentile values proposed by Kułaga et al. [22] with differences reaching up to 11 %. Moreover, underweight was diagnosed three times as often in girls and two times in boys when applying the IOTF standard in comparison to the criteria developed by Tomaszewski et al. [24]. In contrast, the application of those same criteria highlighted a slight increase in the occurrence of obesity and overweight in boys than other methods, which correlates with the results described in the previous study [24]. Similar observations have been made by other researchers [46, 47] who stated that IOTF obesity criteria for the European population are not strict enough and may produce inaccurate results in large-scale studies. In the study of Shields and Tremblay [48], the incidence of obesity was markedly lower when Cole’s cut-off points were applied compared with other reference values (8 and 13 %, respectively). The discrepancies between BMI reference values were also reported by Flegal et al. [49] who compared estimates based on various sources [14, 50, 51]. The inconsistencies were probably due to the different approaches used to define cut-offs and to different criteria used to select study samples [52]. In spite of the perceived differences, the results obtained in this study indicated a significant correlation between the assumed standards in terms of the percentage of compliant classifications reaching 90 % and Cohen’s kappa coefficient values exceeding 0.7. Likewise, the assessment of the prevalence of central obesity using the WHtR indicator produced very similar results for both methods (19–21 %) with classification compliance exceeding 91 %. However, it should be emphasized that the application of the criteria proposed by Stupnicki et al. [19] allowed for the diagnosis of abdominal fat distribution in another 7.1 % of girls and 7.5 % of boys, characterized by increased levels of body fat which places them at a high risk of developing the particularly dangerous central obesity.

What is interesting is the results of this study showing considerable discrepancies between BMI- and WHtR-based obesity estimations. Namely, of the girls with WHtR values ≥0.5, only 14–23 % was classified obese while about one third had normal BMI values, depending on the BMI standard used. In boys, the discrepancies were less pronounced; about 30–40 % of participants with WHtR values ≥0.5 were obese using different BMI standards while percentages of those with BMI values within normal limits varied from 8 to 23 %. This indicates that majority of children who were classified as centrally obese were overweight and, therefore, not yet considered obese using BMI standards. On the other hand, irrespectively of the BMI standard used, all participants classified as obese using BMI criteria had WHtR values ≥0.5. Likewise in the study of Goulding et al. [53], the comparison of children above the 95th percentile for BMI and those with WHtR values >0.5 showed good agreement; as many as 90 % of children with BMI-diagnosed obesity had WHtR values >0.5. All aforementioned observations partly confirm findings of Ashwell and Hsieh [40] that WHtR is more sensitive than BMI as an early detection of obesity and obesity-related health risks. However, the results of this study should be interpreted with caution due to the relatively small sample size.

Summing up, the estimated prevalence of underweight, overweight and obesity is with some exceptions comparable to the findings of other Polish studies conducted among urban children. Moreover, a considerably high degree of statistical compliance of all methods applied suggests that employing international standards for the assessment of overweight and obesity in the Polish population of children may be sufficiently effective when making comparisons with other countries and monitoring future trends and changes. On the other hand, criteria for assessing obesity and overweight ought to closely relate to the population of a given country and employ the most recent evaluation standards. In addition, considering the fact that the established norms should reflect the recommended standards rather than the present conditions, it appears rational to employ reference values calculated on the basis of statistical data gathered from the population with healthy body fat percentage values, which is a reliable indicator for diagnosing obesity.

## Conclusions

The results of this study indicate that less than 10 % of 7-year-old to 10-year-old urban children were underweight, 15 % of which were diagnosed as overweight and 5 % as obese, which correlates with the statistical rates of the general population. Of particular concern is the rate of occurrence of central obesity, which affects one out of every five children with another 7 % being at risk of developing central obesity. Although the estimates determined according to various standards indicated a high degree of statistical compliance, it seems much more reasonable to apply reference values based on the most effective criteria for diagnosing obesity: the body fat percentage. Adopting these criteria would allow for a more efficient and definitive diagnosis of obesity, especially the particularly dangerous central obesity.

## Abbreviations

%FAT: body fat percentage
BMI: body mass index
IOTF: International Obesity Task Force
LMS: lambda-mu-sigma
SD: standard deviation
UNICEF: United Nations Children’s Fund
WC: waist circumference
WHO: World Health Organization
WHtR: waist-to-height ratio
